# Soil microbial community shifts explain habitat heterogeneity in two *Haloxylon* species from a nutrient perspective

**DOI:** 10.1002/ece3.9727

**Published:** 2023-01-03

**Authors:** Chenhua Li, Yan Li, Lisong Tang, Makoto Ikenaga, Ran Liu, Guiqing Xu

**Affiliations:** ^1^ State Key Laboratory of Desert and Oasis Ecology, Xinjiang Institute of Ecology and Geography Chinese Academy of Sciences Urumqi Xinjiang China; ^2^ Fukang Station of Desert Ecology Chinese Academy of Sciences Fukang Xinjiang China; ^3^ Univerisity of Chinese Academy of Sciences Beijing China; ^4^ Research Field in Agriculture, Agriculture Fisheries and Veterinary Medicine Area Kagoshima University Kagoshima Japan

**Keywords:** desert soils, habitat heterogeneity, microbial community, rhizosphere, sister taxa

## Abstract

*Haloxylon ammodendron* and *Haloxylon persicum* (as sister taxa) are dominant shrubs in the Gurbantunggut Desert. The former grows in inter‐dune lowlands while the latter in sand dunes. However, little information is available regarding the possible role of soil microorganisms in the habitat heterogeneity in the two *Haloxylon* species from a nutrient perspective. Rhizosphere is the interface of plant–microbe–soil interactions and fertile islands usually occur around the roots of desert shrubs. Given this, we applied quantitative real‐time PCR combined with MiSeq amplicon sequencing to compare their rhizosphere effects on microbial abundance and community structures at three soil depths (0–20, 20–40, and 40–60 cm). The rhizosphere effects on microbial activity (respiration) and soil properties had also been estimated. The rhizospheres of both shrubs exerted significant positive effects on microbial activity and abundance (e.g., eukarya, bacteria, and nitrogen‐fixing microbes). The rhizosphere effect of *H. ammodendron* on microbial activity and abundance of bacteria and nitrogen‐fixing microbes was greater than that of *H. persicum*. However, the fertile island effect of *H. ammodendron* was weaker than that of *H. persicum*. Moreover, there existed distinct differences in microbial community structure between the two rhizosphere soils. Soil available nitrogen, especially nitrate nitrogen, was shown to be a driver of microbial community differentiation among rhizosphere and non‐rhizosphere soils in the desert. In general, the rhizosphere of *H. ammodendron* recruited more copiotrophs (e.g., Firmicutes, Bacteroidetes, and Proteobacteria), nitrogen‐fixing microbes and ammonia‐oxidizing bacteria, and with stronger microbial activities. This helps it maintain a competitive advantage in relatively nutrient‐rich lowlands. *Haloxylon persicum* relied more on fungi, actinomycetes, archaea (including ammonia‐oxidizing archaea), and eukarya, with higher nutrient use efficiency, which help it adapt to the harsher dune crests. This study provides insights into the microbial mechanisms of habitat heterogeneity in two *Haloxylon* species in the poor desert soil.

## Graphical abstract

*Haloxylon ammodendron* and *Haloxylon persicum* (as sister taxa) grow in adjacent and distinct habitats in the Gurbantonggut Desert, with the former growing on interdunes and the latter on sand dunes. The rhizosphere of *H. ammodendron* recruited more copiotrophs (e.g., Proteobacteria, Bacteroidetes, N‐fixing microbes, and AOB), and with stronger microbial activities, which helps it maintain a competitive advantage in relatively nutrient‐rich lowlands. *H. persicum* relied more on eukarya, fungi, actinomycetes, and archaea (including AOA), with higher nutrient use efficiency, which help it adapt to the poorer sand dunes.

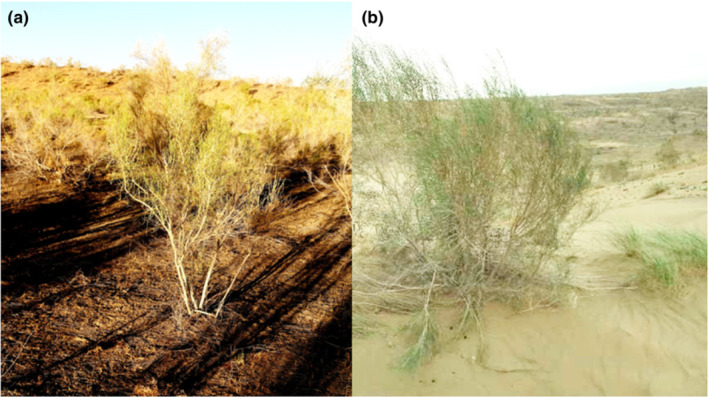

## 1 INTRODUCTION

In arid and semi‐arid ecosystems, shrubs create high‐nutrient patches and spatial variability of soil properties in the low‐nutrient matrix, termed “fertile islands” (Cao et al., 2016; Diedhiou‐Sall et al., 2013). By increasing soil moisture and protecting understory soils from the effects of high temperature, shrubs help soils retain nitrogen (N), increase soil organic matter, and create local microsites for microorganisms (MacMahon & Wagner, 1985). These environmental modifications improve soil microbial communities via increased biomass and activity (Jia et al., 2010). In turn, shrubs stabilize sand dunes by forming fertile islands, which are ecologically and economically important in desert ecosystems (Cao et al., 2016). As a result, the spatial distribution of desert shrubs has a pronounced influence on the biogeochemical cycles of soil nutrients (Cross & Schlesinger, 1999).

The interactions between plants and microorganisms are essential for maintaining plant function and ecological niche (Díaz‐Muñoz, 2017). Most desert microbial communities appear to be maintained primarily via abiotic processes (Cary et al., 2010), since deserts represent extreme environments that result in low microbial diversity (Neilson et al., 2012). However, perennial shrubs maintain a tight internal cycle in deserts, where they readily absorb, transport, and recycle nutrients (Soussi et al., 2016). The truth is that root‐associated microbiota contribute to such adaptations (Mukhtar et al., 2021). The presence or emergence of desert plants, especially shrubs, will inevitably affect the diversity of soil microbial communities, due to the priming effect from the plant rhizosphere. This is demonstrated by the greater soil microbial activity (i.e., CO_2_ efflux) in soils that contain plant materials than those in bare (nonplant) soils (Dijkstra et al., 2009). Recent studies have also shown that soil microbial diversity, rather than plant diversity, is a key limiting factor affecting ecosystem functioning and stability in high‐aridity regions (Hu et al., 2021). Although the biomass of soil microorganisms in desert areas is small, they are still highly active and play a significant role in promoting plant health (Köberl et al., 2011).

*Haloxylon ammodendron* (C.A.Mey.) Bunge and *H. persicum* Bunge ex Boiss. et Buhse (Chenopodiaceae) are sister taxa in the *Haloxylon* genus of Chenopodiaceae family. They are found mainly in the Middle East, Central Asia, Afghanitsan, and Iran (Pyankov et al., 1999) and are the dominant perennial shrubs in the Gurbantunggut Desert, center of the Eurasian Continent. *H. ammodendron* grows at the bottom of desert sand dunes, while *H. persicum* mainly grows along dune crests. The species are thus largely spatially segregated within the same ecosystem, and both play an important role in maintaining ecological stability of the desert (Xu et al., 2007). These two *Haloxylon* species from arid regions are highly water limited, and as a result, the habitat differentiation between *H. ammodendron* and *H. persicum* is explored mainly from the aspect of plant–water relationships (Dai et al., 2015). The focus on water use, however, may mask the additionally important role of nutrient limitation in desert plants. Soil nutrients are also highly limiting in desert ecosystems, and thus likely have an important influence on plant species composition (Huang et al., 2018). But so far, knowledge of nutrient availability and plant‐associated microbial communities in the desert soil remains fragmentary and scarce.

Rhizosphere, as the interface of plant–microbe–soil interactions, is crucial to the regulation of soil carbon and nitrogen biogeochemical cycles (Pathan et al., 2020). As a result, it is the most important area for plants to absorb soil nutrients during their growth and development. Rhizosphere effects in arid deserts are more significant than that in farmland and forest soils because of the lower nutrient content of the soil. Likewise, the nutrient interception in the rhizosphere is also stronger in desert soil (Huang et al., 2016). The characteristics of rhizosphere soils may thus be one of the most direct ways in which desert plants utilize nutrients and adapt to harsh environment (Cao et al., 2016; Shmueli et al., 2007). Therefore, comparing the rhizosphere effects of two *Haloxylon* species on soil chemical and microbial properties can better understand the possible role of soil microorganisms in their habitat heterogeneity from a nutrient perspective.

This study thus focuses on the rhizosphere soils (0–60 cm) of *H. ammodendron* and *H*. *persicum* and their corresponding soil environments in the Gurbantunggut Desert. Given that N is the most crucial limiting nutrient factor for plant growth in arid area (Huang et al., 2016), we applied quantitative real‐time PCR (qPCR) to evaluate the variations in the abundance of bacteria, eukarya, archaea, and N‐transforming microorganisms (N‐fixing microbes, ammonia‐oxidizing bacteria, and ammonia‐oxidizing archaea), as affected by the habitat and rhizosphere. Meanwhile, the study also evaluated compositional shifts in bacterial and eukaryal communities using MiSeq amplicon sequencing. On this basis, we compared rhizosphere effects between these two shrubs in term of microbial activity (i.e., respiration), abundance and community structure, as well as for soil properties. The objective of this study was to detect the relationship between soil microbes and the two *Haloxylon* species in adjacent and distinct niches from a nutrient perspective. We hypothesized that (1) The rhizosphere priming effect of *H. ammodendron* and *H*. *persicum* on microbial respiration are different and is related to the recruitment of microorganisms to the rhizosphere and (2) microbial communities in rhizosphere soils of the two shrubs should be clearly differentiated, which corresponds to the soil environmental conditions, especially nutrient availability.

## 2 MATERIALS AND METHODS

### 2.1 Site description

**FIGURE 1:**
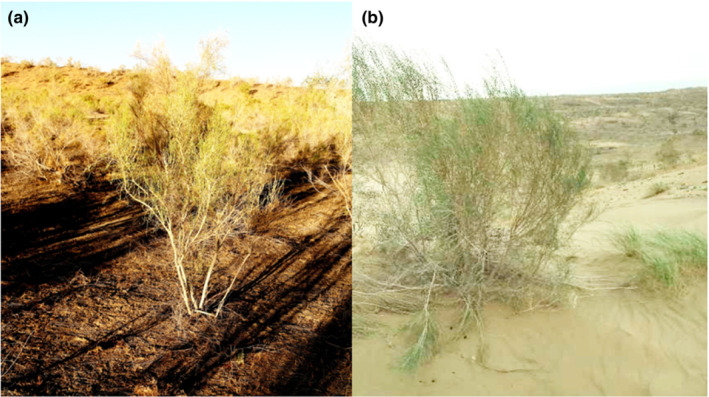
*Haloxylon ammodendron* growing on interdune lowlands (a) and *Haloxylon persicum* growing on sand dunes (b) in the Gurbantunggut Desert, center of the Eurasian continent

The study was conducted within the vicinity (44°17′ N, 87°56′ E, and 475 m a.s.l.) of the Fukang Station of Desert Ecology, Chinese Academy of Sciences. The site lies along the southern edge of the Gurbantunggut Desert. This region is a temperate desert with an arid continental climate that has a cold winter and dry hot summers. The annual mean temperature is 6.6°C. The annual mean precipitation is 160 mm, of which 25% is generally snowfall. Potential annual evaporation is 900 mm (Dai et al., 2015). Dendritic and longitudinal sand dunes characterize the whole landscape. The sand dunes reach a height of 5 to 12 m. The zonal soils are Torripsamments with loamy fine sand texture (81.7% sand, 16.8% silt, and 1.5% clay for the inter‐dunes). Compared with inter‐dunes, dune crests have higher sand content (>95%) and lower nutrient content (Dai et al., 2015; Xie et al., 2015). The desert area is either covered mainly with shrubs and semi‐shrubs or bare soil. These plant communities are poor in species, and most of them were single‐layer structure of low coverage (Xu et al., 2007). Although *H. ammodendron* and *H*. *persicum* grow in adjacent and distinct habitats (inter‐dunes and dune crests) in the desert (Figure 1), they (as sister taxa) share some similarities in morphology, deep root system, tree life forms, and photosynthetic pathway of C4 (Pyankov et al., 1999; Xu et al., 2007; Zou et al., 2010). The densities of adult plants are 700 and 120 plants ha^−1^ in the habitats of *H. ammodendron* and *H. persicum*, respectively (Dai et al., 2015).

Snowfall events in the region occur primarily between January and March, with snow cover reaching a depth of 20–30 cm. The combined inputs of snow melt and rainfall lead to the highest soil moisture content in spring (from April to May), which provides plenty of water for the germination and growth of desert plants (Zhou et al., 2009). During this period, shallow soil water is the main source of water used by both *H. ammodendron* and *H*. *persicum* (Dai et al., 2015). Likewise, the rhizosphere priming effect (i.e., decomposition of organic matter by microorganisms) is also strongest during this stage (Warembourg & Estelrich, 2001). The groundwater table depth is more than 4 m.

### 2.2 Soil sampling and chemical analysis

Soil sampling was performed in the native habitats for *H. ammodendron* and *H. persicum* in early May 2019, coinciding with rapid growth in these species. A typical representative sand dune and the corresponding inter‐dune site were chosen along the southern edge of the Gurbantunggut Desert. The dune crest was about 11 m higher than the inter‐dune lowland. Three mature individual shrubs for each species were randomly selected in the sand dune site and inter‐dune site with about the middle age, similar height and basal stem diameters and canopy size. The average height, basal diameters, and canopy radii were 200–215 cm, 9.1–11.0 cm, and 1.04–1.11 m for *H. ammodendron*, respectively, and 260–285 cm, 8.3–10.1 cm and 1.10–1.18 m for *H*. *persicum*. The selected individual shrubs were at least 5 m away from the surrounding shrubs to exclude the effect of plant densities on rhizosphere soil sampling. Given that the roots of desert shrubs that absorb shallow soil moisture are mainly distributed at the first 60 cm depth of soil profile, and the sampling depth was set at 0–60 cm (Xu et al., 2007). Meanwhile, three rhizosphere soil samples around each individual shrub were collected randomly in each site. We selected three sampling depths: 0–20, 20–40, 40–60 cm, mainly based on the differences in moisture and compactness along the soil profile (0–60 cm) and the sampling depths in the previous study (Zou et al., 2010). Samples taken at the same depth for each individual shrub were mixed to obtain a representative soil sample. Meanwhile, three sampling points also were randomly picked out in the interplant (bulk soils) both for the dune crest site and inter‐dune site.

Next, soil samples were divided into three parts after removal of visible plant root and organisms. One part was stored at 4°C to measure microbial activity within 2 weeks; one was frozen at −80°C for analysis of microbial gene sequences; the remaining samples were air‐dried and passed through a 2‐mm sieve for determination of soil chemical properties. Generally, the fertilizer island effect is characterized by higher organic matter, total N (TN), and available nutrients in soils beneath than outside of shrub canopy (Maestre et al., 2009; Rong et al., 2016). In this study, soil properties, including water content (SWC), pH, electrical conductivity (EC), organic carbon (SOC), TN, available N (AN), and available phosphorus (AP) were determined with the methods described by Li et al. (2019). Extractable ammonium and nitrate‐nitrogen (NH_4_^+^–N and NO_3_^−^–N) contents were determined by the AA3 flow injection analyzers (FIA SFA CFA).

### 2.3 Microbial activity, soil DNA extraction, and quantitative PCR

Soil respiration, which implies the intensity of SOC mineralization, was used as an indicator of microbial activity, and was measured using the alkali absorption method (Menyailo et al., 2003). After adjusting to 40% water‐holding capacity, soil samples were conditioned at 25°C for 4 days in a thermostat‐controlled container. Two beakers containing 10 ml of 1 M NaOH and deionized water were placed in the container to trap released CO_2_ and keep the soil moist. Total CO_2_ efflux was measured using back‐titration with a standardized 0.2 M HCL solution.

Soil DNA was isolated from a minimum of 0.5 g of dry weight soil (DWS) using a FastDNA spin kit (MP Biomedicals) and following the manufacturer's instructions. The integrity of the DNA extracts was confirmed by 0.8% agarose gel electrophoresis in 0.5TBE buffer. Moreover, the extracts were checked for quantity and quality using a Nanodrop ND‐1000 UV‐Vis Spectrophotometer (Nanodrop Technologies). By using quantitative real‐time PCR (qPCR; Table 1), the target genes of bacteria, archaea, eukarya, ammonia‐oxidizing archaea (AOA), ammonia‐oxidizing bacteria (AOB), and N‐fixing microbes were analyzed to quantify their copy numbers. The recombinant plasmids were obtained from the positive clone with the correct DNA insert using a Miniprep kit (Qiagen). The plasmid concentration was determined by a NanoDrop ND‐1000 Spectrophotometer (NanoDrop Technologies). The standard curves of each gene were then obtained by diluting the recombinant plasmids with a 10‐fold gradient. The melt‐curve analysis at 65°C to 95°C (increment of 0.5°C) was performed to confirm PCR production specificity after amplification. Absolute copy number of the target gene was calculated from the plasmid DNA standard curve to generally estimate the abundance of specific microbial community (Okano et al., 2004). The negative control (sterilized ddH_2_O instead of template DNA) was set in qPCR. Meanwhile, the extracted DNA from each soil sample with the same amount of dry soil was amplified in triplicate as a template, to estimate gene copy number differences across different samples. The SYBR® Premix ExTaq TM (Perfect Real Time) kit was used for the quantitative analysis on a CFX96 Real‐Time PCR System (Bio‐Rad). The qPCR system was 20 μl, including 1 μl DNA template diluted 10 times, 10 μl SYBR Premix Ex TAQ TM Perfect Real Time, 0.2 μl (20 mol L^−1^) primer and 8.6 μl sterile double distilled water.

**TABLE 1:** Details regarding the primers and PCR conditions used for quantitative PCR in this study

Taxa	Taget genes	Primer sequences (5′‐3′)	Length of amplicons	Thermal profile for PCR
Bacteria	16 S rRNA	515F: GTGCCAGCMGCCGCGG	382 bp	95°C, 3 min; 40× (95°C, 30 s; 55°C, 30 s; 72°C, 30 s; with plate read)
		907R: CCGTCAATTCMTTTRAGTTT Lane (1991)		
Archaea	16 S rRNA	364aF: GGGGYGCASCAGGCGCGAA	570 bp	95°C, 3 min; 40× (95°C, 30 s; 58°C, 30 s; 72°C, 30 s; with plate read)
		934bR: GTGCTCCCCCGCCAATTCCT Kemnitz et al. (2005)		
Eukarya	18 S rRNA	Euk1A: CTGGTTGATCCTGCCAG Euk516R: ACCAGACTTGCCCTCC Díez et al. (2001)	560 bp	95°C, 3 min; 40× (95°C, 30 s; 56°C, 30 s; 72°C, 30 s; with plate read)
AOA	amoA	Arch‐amoAF: TAATGGTCTGGCTTAGACG	635 bp	95°C, 30 s; 40× (95°C, 5 s; 55°C, 30 s; 72°C, 30 s; with plate read)
		Arch‐amoAR: CGGCCATCCATCTGTATGT Francis et al. (2005)		
AOB	amoA	amoA‐1F: GGGGTTTCTACTGGTGGT	491 bp	95°C, 30 s; 40× (95°C, 5 s; 55°C, 30 s; 72°C, 30 s; with plate read)
		amoA‐1R: CCCCTCKGSAAAGCCTTCTTC Rotthauwe et al. (1997)		
N‐fixing microbes	nifH	POIF: TGCGAYCCSAARGCBGACTC	361 bp	95°C, 10 min; 40× (95°C, 30 s; 55°C, 30 s; 72°C, 30 s; 80°C, 5 s; with plate read)
		POIR: ATSGCCATCATYTCRCCGGA Poly et al. (2001)		

### 2.4 16 S and 18 S rRNA gene pyrosequencing

The lichen soil crusts are common in the desert ecosystem (Huang et al., 2018). Lichens contain key members such as algae and microbes. As pioneers in desert and semi‐desert ecosystems, they play a key role in the formation of desert soil and the colonization of vascular plants by increasing soil C, N, and P contents (Zhou et al., 2016). Thus, while the soil bacterial community was analyzed, the eukaryal community was also analyzed to estimate changes in both fungal and algae communites in this study.

Accordingly, both 16 S and 18 S rRNA gene pyrosequencings were performed on a Miseq System sequencer (Illumia, Inc.). Briefly, DNA extracts from soil samples were used as templates for PCR amplification after quality control. Specific primers were tagged with unique, sample‐specific barcodes before amplification. PCR was carried out with the 515F and 907R primers (Stubner, 2002) for the amplification of the V3–V4 region of the 16 S rRNA gene from bacteria and archaea. PCR reactions were prepared in a 50 μl mixture containing 4 μl of dNTP Mixture (2.5 mM each), 1 μl of each primer at 10 μM, 1 μl of DNA template, 5 μl of 10× Ex Taq Buffer (Mg^2+^Plus), 0.25 μl of TaKaRa Ex Taq HS at 5 U/μl (TaKaRa Biotech). The PCR program was as follows: 94°C for 5.0 min; 30 cycles of 94°C for 30 s; 55°C for 30 s; 72°C for 45 s; 72°C for 8 min; and hold at 4°C. Meanwhile, soil genomic DNA was also used to amplify 18 S rRNA genes for the eukaryal community by forward primer EUK528FF (GCGGTAATTCCAGCTCCAA) and the reverse primer Euk706R (AATCCRAGAATTTCACCTCT; Cheung et al., 2010). PCR reactions were performed in a 50 μl mixture containing 200 μM of dNTP, 2 μl of DNA template, 0.2 μM of each primer, 5 μl of 10× Ex Taq Buffer (Mg^2+^Plus), and 0.5 μl of TaKaRa Ex Taq HS at 5 U/μl (TaKaRa Biotech). The PCR program was 95°C for 5.0 min; 30 cycles of 95°C for 45 s; 55°C for 45 min; 72°C for 1 min; 72°C for 10 min; and hold at 4°C. Each sample was amplified in triplicate. Sterilized water instead of soil DNA extraction was used as negative controls to check for contamination of primer or sample DNA. The specific adapters required for pyrosequencing were ligated to the 5′ and 3′ ends of the PCR products. After being examined by 1.2% agarose gel electrophoresis, the PCR products in triplicate were pooled and purified using the quantitative DNA binding method (Invitrogen) and quantified with Picogreen (Invitrogen). The purified PCR amplicons with known concentrations were combined in equimolar ratios into a single tube in preparation for pyrosequencing analysis.

Next, the raw sequence reads were processed with the Quantitative Insights Into Microbial Ecology (QIIME version: 1.3.0) according to Caporaso et al. (2010). At the initial steps, the multiplexed reads (i.e., sequences obtained from DNA fragments) were assigned to samples by sample‐specific tag sequences (barcodes). Meanwhile, quality filtering of the reads was performed according to the characteristics of each sequence. After identifying and removing the impurities and low‐quality sequences (quality score < 20), sequences >200 bp in length (quality score > 25), and without ambiguous base calls or mismatches were retained in sequencing analyses (Caporaso et al., 2010). These remaining high‐quality sequences were then clustered into operational taxonomic units (OTU) using the threshold of 97% identity with the UCLUST algorithm (Edgar, 2010). Meanwhile, the most abundant reads from each OTU were selected as the representative sequence of that OTU, which hence are more likely to be correct biological sequences. Taxonomic identity of the representative sequence from each OTU was determined using the Ribosomal Database Project (RDP) Classifier (http://rdp.cme.msu.edu/). The RDP Classifier assigns complete taxonomic information to each sequence in the database with 80% taxonomy confidence (Wang et al., 2007). Good's coverage, as an estimator of sampling completeness, was calculated at a 97% similarity cutoff (Claesson et al., 2009). The taxonomic assignments were used to construct an OTU table, which was a matrix of OTU abundance for each sample with specific taxonomic identifiers for each OTU (Suleiman et al., 2013). Meanwhile, the OTU sequence number was converted into the proportion of the OTU in the sample (i.e., the relative abundance) for cross comparison among the samples. A summary for pyrosequencing results targeting 16 S and 18 S rRNA genes in the sand dune site and inter‐dune site was showed in Table 2.

**TABLE 2:** Summary of pryosequencing results targeting 16 S and 18 S rRNA genes of the microbial community in the two rhizospheres of *Haloxylon ammodendron* (Ha) and *H. persicum* (Hp) and their corresponding bulk soils (Ba for *H. ammodendron* and Bp for *H. persicum*) at 0–20, 20–40, and 40–60 cm depths

	16 S rRNA gene sequencing				18 S rRNA gene sequencing			
	SS	ON	OSN	Coverage	SS	ON	OSN	Coverage
Ba (0–20 cm)								
1	44,714	2954	43,653	0.86	47,018	792	45,573	0.82
2	40,595	2779	38,907	0.83	45,039	535	42,733	0.84
3	36,986	2667	35,370	0.82	42,657	444	41,376	0.80
Ba (20–40 cm)								
1	36,216	2475	34,976	0.83	23,625	603	21,232	0.81
2	31,859	2256	30,601	0.85	22,078	434	19,483	0.80
3	30,882	2056	29,071	0.81	20,265	329	18,546	0.75
Ba (40–60 cm)								
1	49,005	2035	47,523	0.78	6630	253	5157	0.80
2	44,759	1817	43,427	0.80	5961	161	4794	0.81
3	41,975	1656	40,465	0.80	5544	137	4539	0.83
Ha (0–20 cm)								
1	33,983	2006	32,335	0.76	31,347	596	30,295	0.87
2	30,192	1714	28,631	0.75	30,536	371	29,161	0.85
3	29,053	1550	27,495	0.78	30,005	324	28,477	0.80
Ha (20–40 cm)								
1	38,357	2503	37,207	0.84	16,398	535	15,382	0.83
2	36,669	2353	35,288	0.81	16,093	365	14,546	0.78
3	35,754	2.151	34,144	0.81	15,563	333	14,222	0.81
Ha (40–60 cm)								
1	47,353	2365	46,289	0.90	3696	309	3225	0.83
2	42,936	2275	41,506	0.86	3623	204	2853	0.79
3	39,227	2122	37,960	0.85	3436	164	2651	0.78
Bp (0–20 cm)								
1	29,755	2492	28,336	0.80	8489	437	7991	0.78
2	27,292	2369	25,907	0.81	7662	305	7298	0.81
3	25,165	2227	24,004	0.83	7325	225	6798	0.76
Bp (20–40 cm)								
1	40,254	2862	38,524	0.81	10,369	511	8887	0.80
2	36,631	2763	35,199	0.83	8731	363	8275	0.81
3	34,064	2597	32,800	0.85	8336	341	7869	0.82
Bp (40–60 cm)								
1	19,785	2059	18,271	0.82	22,508	443	21,019	0.83
2	17,533	1895	16,064	0.82	21,682	286	20,509	0.85
3	15,810	1725	14,630	0.80	21,657	253	20,123	0.81
Hp (0–20 cm)								
1	39,281	2761	37,956	0.83	32,180	718	30,954	0.86
2	36,175	2646	34,900	0.85	31,635	558	30,287	0.83
3	34,282	2569	32,709	0.81	30,964	517	29,698	0.83
Hp (20–40 cm)								
1	42,923	2766	41,733	0.84	59,606	735	58,291	0.80
2	39,884	2662	38,347	0.81	58,964	562	57,534	0.82
3	37,795	2539	36,053	0.78	58,213	444	57,057	0.83
Hp (40–60 cm)								
1	22,864	2112	21,557	0.84	10,533	454	9383	0.84
2	20,592	1999	19,328	0.85	10,084	362	8869	0.81
3	19,057	1844	17,746	0.82	10,003	327	8819	0.82

Abbreviations: ON, OTUs number; OSN, OTUs sequence number; SS, sample size.

### 2.5 Data and statistical analyses

Rhizosphere effects on soil chemical and microbial properties were measured as the root soil (R/S) ratio. Statistical analyses were performed using SPSS 20.0 for Windows (SPSS Inc). Analysis of variance (ANOVA) and a Duncan's‐test were used to assess significant differences (*p* < .05) in soil properties, microbial activity, gene copy number, and microbial relative abundances among rhizospheres of the two species and their corresponding bulk soils, after the data were checked for normality and homogeneity of variance. A two‐way ANOVA was used to evaluate the effects of topography and rhizosphere on microbial activity and gene abundance. The Pearson correlation was used to test the significance of correlations between soil properties, microbial activity, and gene copy number.

Redundancy analysis (RDA) was performed to evaluate the relationship between soil properties and bacterial and fungal taxa. It was also used to fully assess changes in microbial community structure across the topography, rhizosphere, and soil depth. Species data were square‐root transformed in the course of this analysis. The corresponding ordination plot was constructed using CANOCO 5.0 for Windows. Nine environmental variances (SWC, pH, EC, SOC, TN, AN, AP, NH_4_^+^–N and NO_3_^−^–N) were identified by the forward‐selection procedure based on the Monte Carlo test (499 permutations) in this analysis. The procedure returns a *p* value concerned with the effect of the environmental variance on microbial community composition.

## 3 RESULTS

### 3.1 Soil properties

The content of SOC and nutrient generally decreased with soil depth, except for the uniform distribution of NH_4_^+^‐N throughout the soil profile (Table 3). Meanwhile, soil nutrient content of inter‐dunes was substantially higher than that for dune crests throughout the soil profile (0–60 cm). The content of SOC and total N and nutrient availability were higher in inter‐dunes than that in dune crests (*p* < .05), but there was no significant difference in NH_4_^+^‐N content between the two sites. Moreover, the rhizosphere soil nutrient content of *H. ammodendron* in inter‐dunes was also generally higher than that for *H. persicum* growing on sand dunes. Compared with the corresponding nonrhizosphere soils, rhizosphere soils for the two shrubs showed greater nutrient and water availability. Specifically, SOC, AN, NO_3_^−^‐N, and SWC were all significantly higher (*p* < .05), except NH_4_^+^‐N and AP. In contrast, soil TN content in the rhizosphere of *H. ammodendron* decreased, but that of *H*. *persicum* increased, compared with nonrhizosphere soils (*p* < 0.05). The R/S ratios of SOC and TN in the soil profile were 1.10–1.60 and 0.64–0.85 for *H. ammodendron* and 1.14–2.15 and 1.50–2.77 for *H. persicum*. In addition, both EC and pH were generally higher on inter‐dunes than on sand dunes, in the rhizosphere than in the non‐rhizosphere and in the rhizosphere of *H. ammodendron* than in that of *H. persicum* (Table 3).

**TABLE 3:** Soil properties [i.e., water content (SWC), pH, electrical conductivity (EC), organic carbon (SOC), total nitrogen (TN), available nitrogen (AN), available phosphorus (AP), ammonium nitrogen (NH_4_^+^–N), and nitrate nitrogen (NO_3_^−^–N)] in the two rhizospheres of *Haloxylon ammodendron* (Ha) and *H. persicum* (Hp) and their corresponding bulk soils (Ba for *H. ammodendron* and bp for *H. persicum*) at 0–20, 20–40, and 40–60 cm depths

	SWC (%)	PH	EC (mS cm^−1^)	SOC (g kg^−1^)	TN (g kg^−1^)	AN (mg kg^−1^)	AP (mg kg^−1^)	NH_4_^+^–N (mg kg^−1^)	NO_3_^−^–N (mg kg^−1^)
0–20 cm									
Ba^a^	2.3 b	8.83 b	0.172 b	1.14 b	0.233 a	16.10 b	9.45 a	2.6 a	5.2 c
Ha	3.3 a	10.1 a	0.470 a	1.69 a	0.148 b	19.00 a	3.54 b	2.5 a	12.6 a
Bp	1.8 c	8.21d	0.133 c	0.34 d	0.035 d	7.71 d	3.85 b	2.6 a	3.3 d
Hp	2.0 bc	8.54 c	0.190 b	0.73 c	0.097 c	8.44 c	3.13 c	2.1 b	6.2 b
20–40 cm									
Ba	2.5 c	9.30 b	0.284 b	0.892 b	0.094 a	13.81 b	3.75 a	2.3 a	10.2 b
Ha	4.3 a	10.12 a	0.607 a	0.981 a	0.080 b	26.83 a	3.23 b	2.1ab	24.2 a
Bp	2.9 c	8.21 d	0.127 d	0.425 d	0.049 c	3.83 d	3.54 ab	2.2 ab	2.2 d
Hp	3.6 b	8.65 c	0.151c	0.484 c	0.082 b	7.79 c	2.82 c	2.0 b	5.9 c
40–60 cm									
Ba	1.5 c	9.29 a	0.932 a	0.475 c	0.096 a	9.81 b	3.02 a	3.0 a	5.4 b
Ha	4.1 a	9.08 a	1.027 a	0.758 a	0.065 b	10.35 a	2.40 b	2.1 b	6.8 a
Bp	2.8 b	8.50 b	0.125 b	0.334 d	0.032 d	9.19 c	2.82 a	2.9 a	4.3 d
Hp	3.1 b	8.50 b	0.155 b	0.584 b	0.048 c	10.45 a	2.40 b	1.5 c	4.9 c

^a^ Treatment means within a depth followed by the same lower case letter are not significantly different (*p* > .05).

### 3.2 Soil microbial activity and abundance

**FIGURE 2:**
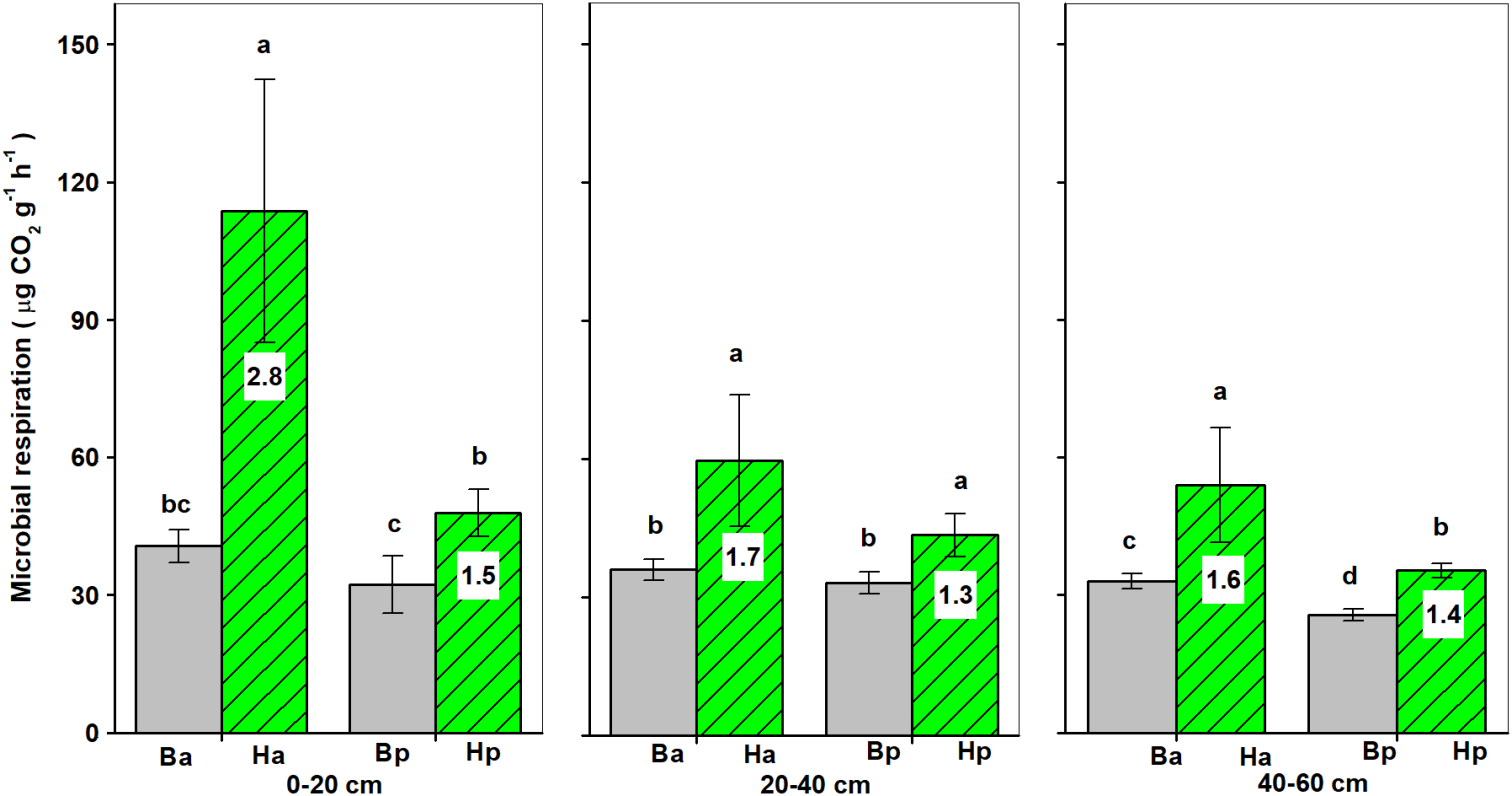
Soil microbial respiration in the two rhizospheres of *Haloxylon ammodendron* (ha) and *H. persicum* (hp) and their corresponding bulk soils (Ba for *H. ammodendron* and Bp for *H. persicum*) at 0–20, 20–40, and 40–60 cm depths. Error bars represent the standard error of the mean (*n* = 3). The numbers in the columns indicate rhizosphere effect (root/soil). Treatment means within a depth followed by the same lowercase letters are not significantly different (*p* > .05)

Soil microbial activity in the rhizosphere of both shrubs was greater than that found in bulk soils (*p* < .05; Figure 2). The rhizosphere effect of *H. ammodendron* on microbial activity was much stronger in topsoils (0–20 cm) than in subsoils (20–60 cm; *p* < .05), while the rhizosphere effect of *H. persicum* was relatively uniform among different soil layers. Moreover, the rhizosphere effect of *H. ammodendron* on microbial activity was significantly stronger than that for *H*. *persicum* (*p* < .05), especially in topsoils.

**FIGURE 3:**
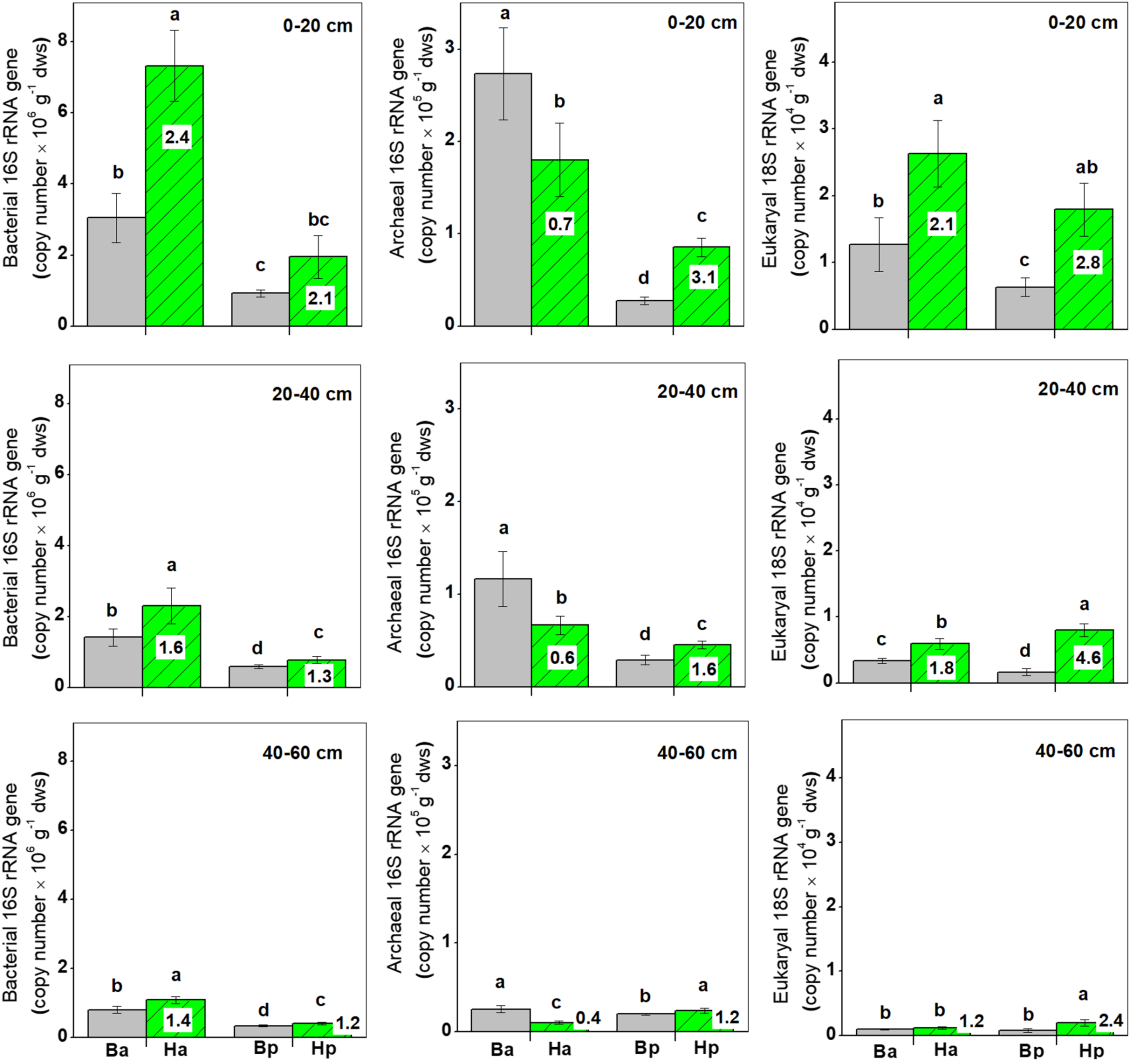
Abundance of bacterial and archaeal 16 S rRNA and eukaryal 18 S rRNA gene copies in the two rhizospheres of *Haloxylon ammodendron* (Ha) and *H. persicum* (Hp) and their corresponding bulk soils (Ba and Bp) at 0–20, 20–40, and 40–60 cm depths. The numbers in the columns indicate rhizosphere effect (root/soil). Treatment means within a depth followed by the same lowercase letters are not significantly different (*p* > .05)

The gene abundance of bacterial and archaeal 16 S rRNA and eukaryal 18 S rRNA substantially decreased with soil depth (Figure 3). Bacterial 16 S rRNA gene abundance throughout the soil profile as well as archaeal 16 S rRNA and eukaryal 18 S rRNA gene abundances at 0–40 cm depths was significantly higher on inter‐dunes than on dune crests (*p* < .05). The gene abundances of bacterial 16 S rRNA and eukaryal 18 S rRNA in the rhizosphere of both shrubs were higher than for bulk soils (*p* < .05). The rhizosphere effect of *H. ammodendron* on bacterial 16 S rRNA gene abundance was greater than that for *H*. *persicum* throughout the soil profile (*p* < .05). However, the stronger rhizosphere effect on eukaryal 18 S rRNA gene abundance was found for *H*. *persicum* than for *H. ammodendron*. In addition, archaeal 16 S rRNA gene abundance significantly decreased in the rhizosphere soil of *H. ammodendron*, but significantly increased in the rhizosphere soil of *H. persicum*, compared with the corresponding bulk soils (*p* < .05).

**FIGURE 4:**
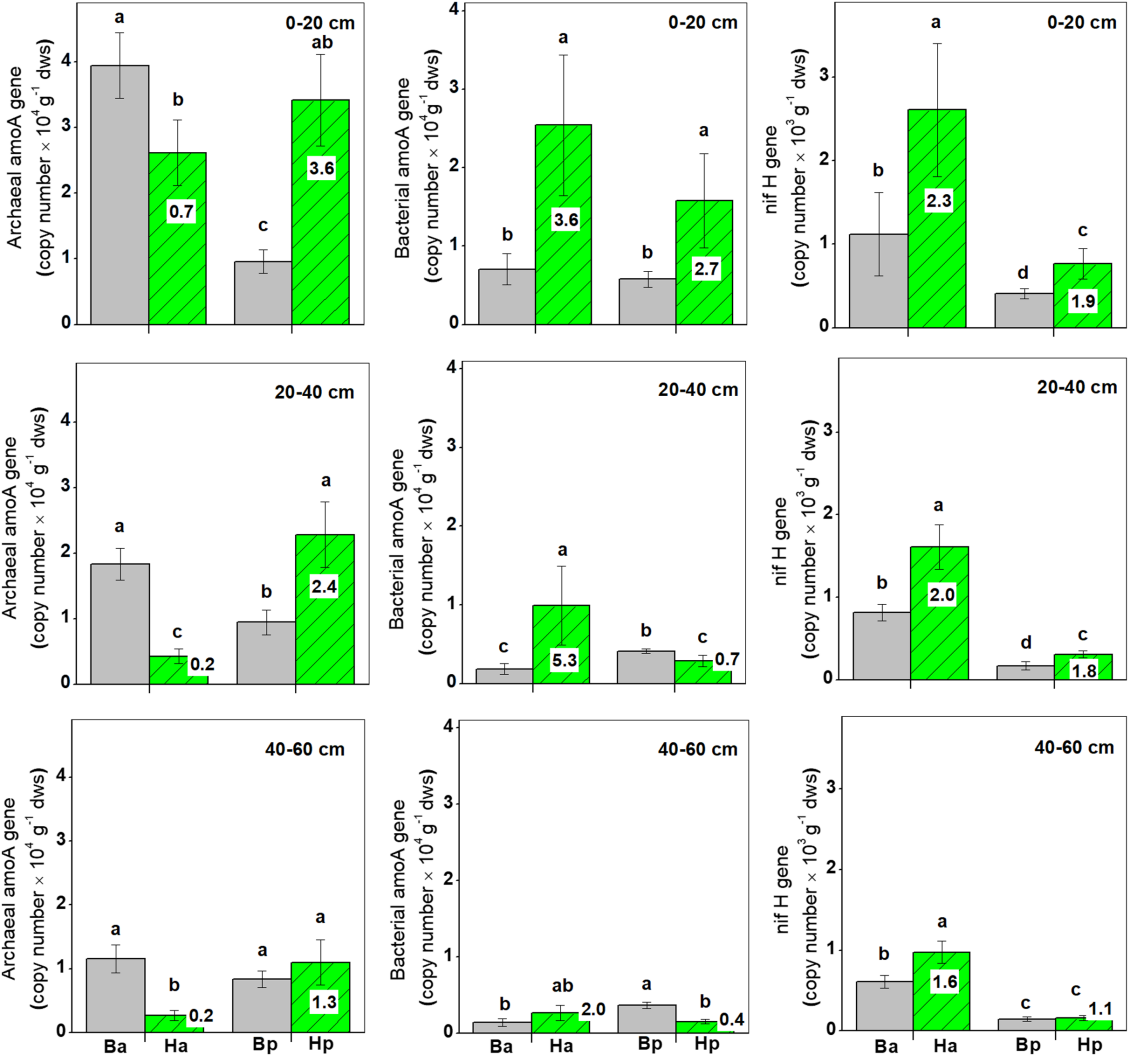
Abundance of archaeal and bacterial amoA and nifH gene copies in the two rhizospheres of *Haloxylon ammodendron* (Ha) and *H. persicum* (Hp) and their corresponding bulk soils (Ba and Bp) at 0–20, 20–40, and 40–60 cm depths. The numbers in the columns mean the rhizosphere effect (root/soil). Treatment means within a depth followed by the same lowercase letters are not significantly different (*p* > .05)

Compared to the corresponding bulk soils, archaeal amoA gene abundance in the soil profile decreased significantly in the rhizosphere of *H. ammodendron*, but increased significantly in the rhizosphere of *H. persicum* (*p* < .05; Figure 4). Meanwhile, bacterial amoA gene abundance increased significantly in the rhizospheres of *H. ammodendron* (throughout the soil profile) and *H. persicum* (in the topsoil; *p* < .05). However, the rhizosphere effect of *H. ammodendron* on bacterial amoA gene abundance was greater than that for *H. persicum*. Moreover, the gene abundance decreased significantly in subsoils of *H. persicum* rhizosphere compared with bulk soils (*p* < .05). In addition, the nifH gene abundance in the rhizosphere of both shrubs increased significantly compared to their bulk soils (*p* < .05), with greater increases for *H. ammodendron*.

### 3.3 Relative abundance of microbial taxa

**FIGURE 5:**
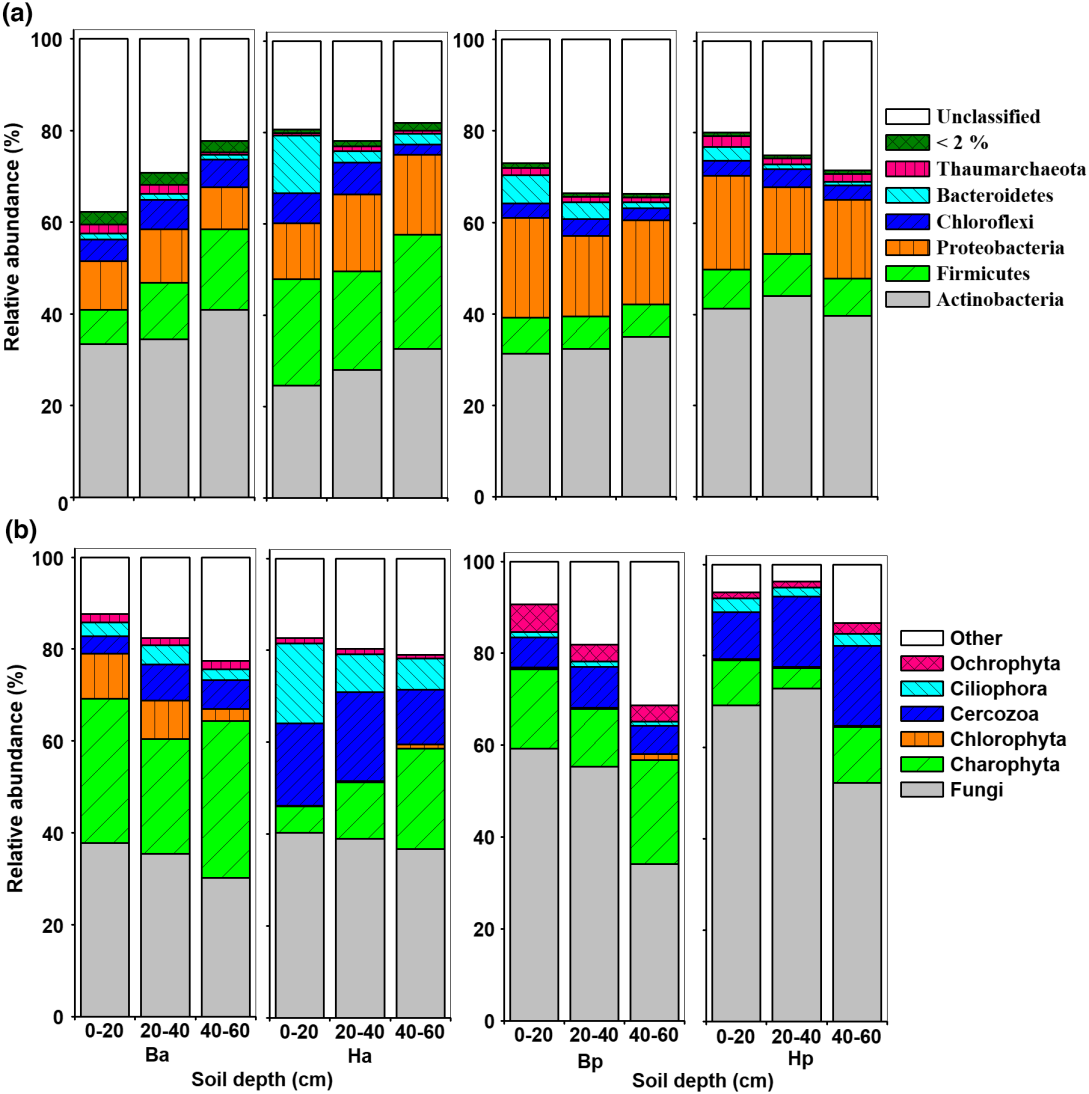
Relative abundances of bacterial and archaeal (a) and eukaryal phyla (b) in the two rhizospheres of *Haloxylon ammodendron* (Ha) and *H. persicum* (Hp) and their corresponding bulk soils (Ba and Bp) at 0–20, 20–40, and 40–60 cm depths

For bacterial communities (Figure 5a), Actinobacteria and Proteobacteria were the predominant phyla in the desert soil. Firmicutes and Chloroflexi were more abundant in inter‐dune soils, while Proteobacteria and Bacteroidetes were more abundant in sand dune soils. Compared with the corresponding bulk soils, the rhizosphere of both shrubs significantly increased the relative abundance of Firmicutes and Proteobacteria. Such increases were greater for *H. ammodendron* than for *H. persicum*. The rhizosphere of *H. ammodendron* also produced higher relative abundance of Bacteroidetes and lower relative abundance of Actinobacteria (*p* < .05). In contrast, the rhizosphere of *H*. *persicum* produced higher relative abundance of Actinobacteria and Chloroflexi and lower relative abundance of Bacteroidetes (*p* < .05). In addition, the Thaumarchaeota, as a novel archaeal phylum, was also more abundant in the rhizosphere of *H. persicum* (*p* < .05).

For eukaryal communities, fungi were dominant members, especially on sand dunes (Figure 5b). Charophyta, Chlorophyta, and Ciliophora were more abundant in inter‐dune soils, and Ochrophyta was more abundant in sand dune soils. Compared with bulk soils, the relative abundance of fungi and protozoa (e.g., Cercozoa and Ciliophora) was higher while microscopic algae were lower in rhizospheres of both shrubs (*p* < .05).

### 3.4 Analyses of variance and correlation

Topography had a significant effect on microbial abundance (e.g., N‐fixing microbes, bacteria, and archaea) and activity (*p* < .05), especially on the abundance of N‐fixing microbes and bacteria and microbial activity (*p* < .001; Table 4). The plant rhizosphere also exerted a significant influence on microbial activity and abundance of eukarya, N‐fixing microbes, AOB, archaea, and bacteria (*p* < .05). In addition, there were significant interactions between rhizosphere and topography on N‐fixing microbe abundance and microbial activity (*p* < .01). In the desert, soil pH was positively correlated with the abundance of bacteria and N‐fixing microbes (*p* < .05), while EC had no significant correlation with microbial abundance (Table 5). The SOC content was significantly positively correlated with the abundance of N‐fixing microbes, bacteria, archaea, eukaryotes, and AOB (*p* < .05), especially with N‐fixing microbes and bacteria (*p* < .01). Soil microbial activity was significantly correlated with the abundance of bacteria, AOB, N‐fixing microbes, and eukaryotes (*p* < .05), especially with N‐fixing microbes (*p* < .01).

**TABLE 4:** ANOVA results regarding microbial abundance and activity (i.e., respiration)

Taxa	Topography		Rhizosphere		Topography×rhizosphere	
	*F*	*p*	*F*	*p*	*F*	*p*
Bacteria	25.844	<.001***	7.599	.010*	2.796	.105
Archaea	6.296	.018*	13.529	.001**	0.610	.441
Eukarya	3.291	.080	25.998	<.001***	0.188	.668
AOA	0.420	.522	0.404	.530	0.212	.523
AOB	0.840	.367	18.011	<.001***	2.337	.137
N‐fixing microbes	66.537	<.001***	19.360	<.001***	7.803	.009**
Respiration	26.905	<.001***	45.287	<.001***	11.999	.002**

*p < .05, **p < .01 and ***p < .001.

**TABLE 5:** Correlation of microbial abundances with soil properties [i.e., pH, electrical conductivity (EC), organic carbon (SOC), total nitrogen (TN), available nitrogen (AN), available phosphorus (AP), ammonium nitrogen (NH_4_^+^–N), and nitrate nitrogen (NO_3_^−^–N)] and microbial activity (i.e., respiration)

	Bacteria	Archaea	Eukarya	AOA	AOB	N‐fixing microbes
pH	0.631*	0.434	0.444	0.149	0.526	0.759*
EC	0.076	−0.186	−0.154	−0.390	−0.029	0.378
SOC	0.946**	0.775*	0.756*	0.481	0.746*	0.926**
TN	0.768*	0.941**	0.587	0.786*	0.407	0.573
AN	0.616*	0.482	0.345	0.058	0.438	0.772*
AP	0.477	0.838*	0.285	0.639*	0.094	0.199
NH_4_^+^–N	0.161	0.178	0.022	0.061	0.057	0.105
NO_3_^−^–N	0.410	0.189	0.234	−0.142	0.391	0.675*
Respiration	0.846*	0.422	0.782*	0.240	0.855*	0.917**

*p < .05 and **p < .01.

**FIGURE 6:**
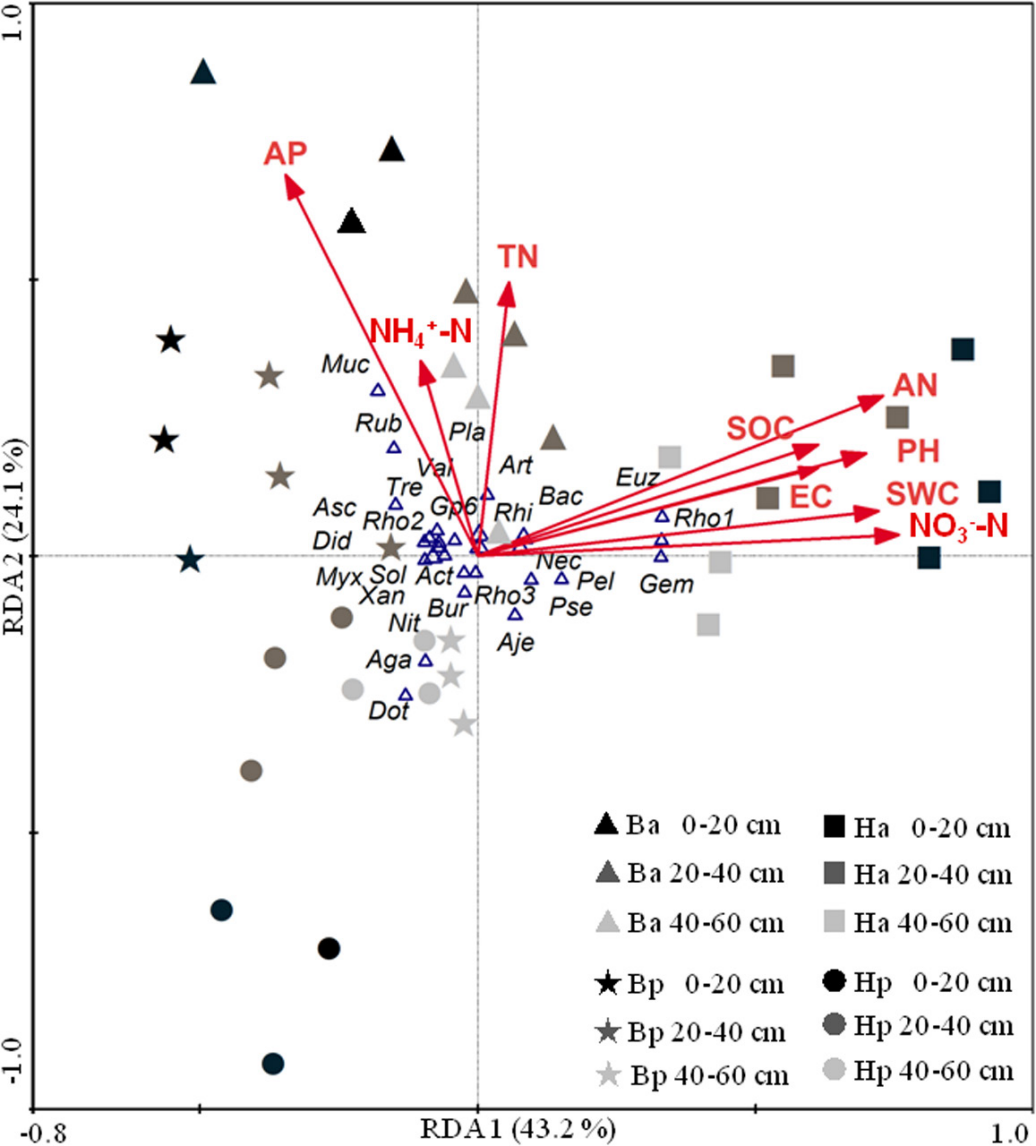
Correlations between microbial community structure and soil properties including soil water content (SWC), pH, electrical conductivity (EC), soil organic carbon (SOC), total nitrogen (TN), available N (AN), available phosphorus (AP), NH_4_^+^‐N, and NO_3_^−^‐N in the two rhizospheres of *Haloxylon ammodendron* at inter‐dune (Ha) and *H. persicum* at sand dunes (Hp) and their corresponding bulk soils (Ba and Bp) at 0–20 20–40, and 40–60 cm depths, as determined by redundancy analysis (RDA). Bacterial orders: Actinomycetales = Act, Bacillales = Bac, Burkholderiales = Bur, Euzebyales = Euz, Myxococcales = Myx, Planctomycetales = Pla, Pseudomonadales = Psu, Rhizobiales = Rhi, Rhodocyclales = Rho1, Rhodobacterales = Rho2, Rhodospirillales = Rho3, Rubrobacterales = Rub, Solirubrobacterales = Sol, Xanthomonadales = Xan. Archaeal orders: Nitrososphaerales = Nit. Fungal families: Agaricaceae = Aga, Ajellomycetaceae = Aje, Arthrodermataceae = Art, Ascobolaceae = Asc, Didymellaceae = Did, Dothideomycetes = Dot, Geminibasidiaceae = Gem, Mucoraceae = Muc, Nectriaceae = Nec, Tremellomycetes = Tre, Peltulaceae = Pel, Valsaceae = Val

We found that most soil chemical properties (except AP, TN, and NH_4_^+^‐N) significantly affected the soil microbial community in the desert (Table 6). The order of influence was AN > NO_3_^−^‐N > SWC > SOC > pH > EC. The RDA ordination plot showed that the relationship between microbial communities and soil chemical properties in four sites (the bulk and rhizosphere soils of *H. ammodendron* on inter‐dunes and *H*. *persicum* on sand crests; Figure 6). The rhizospheres at three depths (0–20, 20–40, and 40–60 cm) for *H*. ammodendron were centered on areas with relatively high moisture, SOC and N content, while the other sites (the rhizospheres of *H*. *persicum* and bulk soils for the two shrubs) were centered along a gradient with lower moisture, SOC, and N content. Meanwhile, the topsoils of four sites were scattered in different areas in the ordination plot (Figure 6). Also, the farthest distance was found between the two shrub rhizospheres. This showed that the microbial community structure between the two rhizospheres had the biggest difference in topsoils. However, the differences in microbial community structure between sample sites decreased with soil depth.

**TABLE 6:** Forward selection of soil variables [i.e., water content (SWC), pH, electrical conductivity (EC), organic carbon (SOC), total nitrogen (TN), available nitrogen (AN), available phosphorus (AP), ammonium nitrogen (NH_4_^+^–N), and nitrate nitrogen (NO_3_^−^–N)] by redundancy analysis with Monte Carlo test

	AN	NO_3_^−^‐N	SWC	SOC	pH	EC	AP	TN	NH_4_^+^‐N
Rank	1	2	3	4	5	6	7	8	9
*F*	3.478	3.328	2.658	2.647	2.618	2.205	1.522	0.855	0.434
*p*	.002**	.008**	.012*	.018*	.022*	.046*	.242	.464	.858

*p < .05 and **p < .01.

## 4 DISCUSSION

### 4.1 Rhizosphere effect of the two *Haloxylon* species

In the Gurbantunggut Desert, early spring ephemeral plants and shrubs grow fastest and absorb the most nutrients in May (Huang et al., 2016). As aboveground biomass increases, soil N concentration decreases, resulting in N limitation due to plant growth (LeBauer & Treseder, 2008). Under soil N stress, root activity and secretion are also enhanced. N limitation could further promote the rhizosphere priming of soil organic matter (Nascente et al., 2021). Rhizosphere microbial respiration is the result of microbial utilization of rhizosphere sediments (Kumar et al., 2016). In the current study, both *Haloxylon* species showed higher SOC, AN, NO_3_^−^‐N and microbial respiration in rhizosphere soils, but reduced NH_4_^+^‐N content. Our results suggest that ammonia oxidation and microbial activity are enhanced in the rhizosphere for both shrub species. This also demonstrates the enhancement of microbial transformations of C and N during the vigorous growth period in the arid desert area. Furthermore, previous studies found that soil C‐N interactions can drive soil N storage (Perroni‐Ventura et al., 2010). In our own study, both shrub species showed enhanced N availability through rhizosphere priming and microbial activation, and, in turn, contribute to the formation of “fertility islands” in the desert.

In this study, the abundance of bacteria, fungi, and N‐fixing microbes also increased in the rhizosphere soils of both *Haloxylon* species, and there was microbial community differentiation between rhizoshere and non‐rhizosphere soils. Rhizospheric microbial communities primarily originate from the surrounding non‐rhizosphere soils (Zarraonaindia et al., 2015). The abscission of plant roots and the secretion of organic matter then provide the necessary nutrients for the microflora. Meanwhile, signal molecules secreted by roots can recruit specific beneficial microfloras (van Dam & Bouwmeester, 2016; Venturi & Keel, 2016). This can partially explain the assembly of plant‐associated microbes and the difference in microbial communities between rhizosphere and non‐rhizosphere soils. In addition, some obligate bacteria can produce a range of bioactive compounds that can promote plant growth (Compant et al., 2010). In this study, we found that *Bacillus* and *Pseudomonas* spp. were more abundant in the rhizosphere soils of both *Haloxylon* species (Figure 6). Both bacterial types are considered highly important at promoting plant growth (Edwards et al., 2015; Vandenkoornhuyse et al., 2015). Our findings indicate that desert plants can still recruit beneficial microorganisms by the rhizosphere, which may be one of the important mechanisms by which they can better adapt and survive in harsh desert environments.

### 4.2 Variation of rhizosphere effects within two *Haloxylon* species

The current study confirms our hypothesis that there were significant variations in rhizosphere effects between the two *Haloxylon* species across different habitats. First, *H*. *ammodendron* showed a much stronger rhizosphere priming effect than did *H. persicum* (Figure 2). In contrast, *H. persicum* had a more significant fertile island effect (higher R/S ratio of SOC and TN content) on the rhizosphere compared to *H. ammodendron* (Table 3). Moreover, the rhizosphere of *H. ammodendron* harbored more copiotrophs (e.g., Firmicutes, Bacteroidetes, and Proteobacteria; Griffith et al., 2017; Oh et al., 2012), N‐fixing microbes and AOB, while the rhizosphere of *H. persicum* harbored more oligotrophs (e.g., actinomycetes; Zhang et al., 2021), archaea (Martens‐Habbena et al., 2009), and fungi, which may be capable of growing as oligotrophs, chemolithoheterotrophs, or even as chemolithoautotrophs (Wainwright, 1988). Meanwhile, this study also showed remarkable differences in microbial community structure between the rhizospheres of the two *Haloxylon* species. For example, the rhizosphere effect on the relative abundance of Bacillales and Pseudomonadales was stronger for *H. ammodendron* in inter‐dunes than for *H. persicum* in dune crests. As important plant growth‐promoting bacteria, these two microbial groups can help root growth (van der Heijden & Schlaeppi, 2015). This corresponds to *H. ammodendron*'s deeper roots, compared to *H. persicum*'s (Dai et al., 2015).

In addition, the significant correlation between microbial activity and specific groups (e.g., N‐fixing microbes and bacteria) demonstrates that soil priming effects are likely produced by rhizosphere‐specific microorganisms in the desert. These results suggest that SOC mineralization and nutrient consumption in the rhizosphere of *H. ammodendron* are stronger than that for *H. persicum*. Meanwhile, *H. persicum* may utilize soil nutrients more efficiently and economically, which would facilitate nutrient storage in the more hostile environment of dune crests. Moreover, such differences in nutrient utilization between the two *Haloxylon* species are closely related to the role of specific microorganisms in their respective rhizospheres.

### 4.3 Factors driving variation in soil microbial communities

Topography significantly affects the distribution of soil water and nutrients, which in turn affects the distribution and growth of plants (Verma & Katpatal, 2021). Sand dunes and interdunes have a profound impact on the distributions of soil water and nutrients across the desert landscape by providing habitat heterogeneity (Dai et al., 2015). Due to stronger wind erosion and light radiation, soil nutrient and water conditions on sand dunes are significantly weaker than those observed on interdunes. Moreover, the plant rhizosphere has an extremely effective enriching effect and seletion effect on soil microbes via eutrophication and difference in soil characteristics caused by the terrain (Lugtenberg & Kamilova, 2009). Our study also confirms that microbial community structure across different ecological niches is the result of environmental filtering in topography and vegetation (Rewald et al., 2014).

Studies have shown that soil nutrients are the main driver of diversity and structural differentiation of soil microbes within the same soil type (Wang et al., 2019). Rhizosphere priming effects are primarily related to N availability, with P availability being less important (Sullivan & Hart, 2013). Our study also supports this finding and suggests the importance of N availability, particularly NO_3_^−^‐N, for plant growth in desert ecosystems (Figure 6). Moreover, there were more AOB and N‐fixing microbes in the rhizosphere of *H. ammodendron* and more AOA in the rhizosphere of *H. persicum*. The results suggest that *H. ammodendron* grows more competitively on interdunes with better nutrient and moisture, while *H. persicum* survives by building its own unique adaptive mechanism in the harsher conditions of dune crests.

The N availability directly affects microbial activity due to the simultaneous assimilation of C and N by microbes (Chen et al., 2019). The present study revealed positive correlations among nifH gene abundance, microbial activity, and soil AN (including NO_3_^−^‐N) and SOC contents (Table 5 and Figure 6). This indicates that in the Gurbantunggut Desert, the rhizosphere of *H. ammodendron* on interdunes is closely related to soil N fixation and SOC mineralization, both of which are mediated mainly by soil bacteria. The rhizosphere of *H. persicum* attracted more fungi and actinomycetes than for *H. ammodendron* (Figure 5). These microbes have higher nutrient use efficiency which likely helps *H. persicum* adapt to more stressful nutrient conditions (i.e., dune crests). Therefore, the assembly of microbial communities in the rhizosphere of the two *Haloxylon* species reflects the heterogeneity of their habitats by varied nutrient utilization strategies.

## 5 CONCLUSION

By focusing on two *Haloxylon* species growing in different habitats of Gurbantunggut Desert, we found important rhizosphere effects on microbial activity, abundance, community, and soil properties. The rhizosphere effects of *H. ammodendron* on microbial activity were stronger than that for *H*. *persicum*, while the former was weaker than the latter when examining the fertile island effect, implying that the rhizosphere soil of *H. persicum* is more favorable for nutrient storage than that of *H. Ammodendron*. We also found significant rhizosphere effects in both shrub species in terms of enhanced abundance of bacteria, eukarya, and N‐fixing microbes. However, the rhizosphere of *H. ammodendron* enriched more bacteria, N‐fixing microbes, and AOB, while the rhizosphere of *H*. *persicum* had higher abundance of eukarya, archeae, and AOA. Meanwhile, the phyla Proteobacteria, Bacteroidetes, and Firmicutes of bacteria were more abundant in rhizosphere soils of *H. ammodendron*, while phyla Actinobacteria and Chloroflexi of bacteria, fungi, and phylum Thaumarchaeota of archaea were more abundant in rhizosphere soils of *H*. *persicum*. Habitat and rhizosphere jointly contribute to differences in soil nutrient status in the desert. The nutrient availability, especially N availability, led to the largest microbial community structure differentiation between the rhizospheres of the two *Haloxylon* species. Our results suggest that *H. ammodendron* grows in interdune lowlands with stronger competitiveness, while *H. persicum* survives better in dune crests of the desert with stronger adaption. This is accomplished partially due to differences in soil microbial community composition as affected by their respective rhizospheres.

## AUTHOR CONTRIBUTIONS

**Chenhua Li:** Data curation (equal); investigation (equal); writing – original draft (lead); writing – review and editing (equal). **Yan Li:** Data curation (equal); supervision (equal); visualization (equal); writing – review and editing (equal). **Lisong Tang:** Funding acquisition (equal); supervision (equal); visualization (equal); writing – review and editing (equal). **Makoto Ikenaga:** Methodology (equal); validation (equal); writing – review and editing (equal). **Ran Liu:** Data curation (equal); investigation (equal); validation (equal). **Guiqing Xu:** Funding acquisition (equal); investigation (equal); validation (equal).

## FUNDING INFORMATION

This work was financially supported by the Special Project on Regional Collaborative Innovation in Xinjiang Uygur Autonomous Region, China (No.2022E01011) and the National Natural Sciences Foundation of China (Nos. 42271068, 32171874 and 42171068).

## CONFLICT OF INTEREST

The authors declare that they have no known competing financial interests or personal relationships that could have appeared to influence the work reported in this paper.

## Data Availability

The data that support the findings of this study will be openly available at Dryad https://doi.org/10.5061/dryad.tdz08kq2p. The raw sequences were submitted to NCBI Sequence Read. Archive database under the BioProject PRJNA907540.
